# The impact of the first 2 years of the COVID-19 pandemic on breast cancer diagnoses: a population-based study in England

**DOI:** 10.1038/s41416-022-02054-4

**Published:** 2022-11-12

**Authors:** Toral Gathani, David Dodwell, Kieran Horgan

**Affiliations:** 1 Cancer Epidemiology Unit, Nuffield Department of Population Health, University of Oxford, Oxford, UK; 2 Department of Breast Surgery, Oxford University Hospitals NHS Foundation Trust, Oxford, UK; 3 Nuffield Department of Population Health, University of Oxford, Oxford, UK; 4 Department of Breast Surgery, Bexley Cancer Centre, St James’s University Hospital, Leeds, UK

**Keywords:** Breast cancer, Cancer epidemiology

## Abstract

Cancer services were seriously disrupted during the COVID-19 pandemic. Using publicly available data for England, we show that breast cancer diagnoses decreased substantially in the first year of the pandemic, but have recovered to at least pre-pandemic levels. Long term follow-up is needed to assess the impact on outcomes.

The impact of the coronavirus disease 2019 (COVID-19) pandemic was significant with three ‘stay at home’ orders of varying durations issued in England in March 2020, November 2020 and January 2021 [1]. During the pandemic, guidance was provided on how to manage and plan the subsequent recovery of cancer services [2].

Breast cancer in England is diagnosed through two main routes largely dependent on patient age. Patients with breast related symptoms and aged ≥16 years may be referred to secondary care breast symptomatic services either urgently, where cancer is suspected, or routinely, where cancer is not suspected [3]. Women aged 50–70 years may also be diagnosed through three yearly mammography within the National Health Service Breast Screening Programme (NHSBSP) [4]. Of the ~50,000 breast cancers diagnosed annually, ~60% of diagnoses follow an urgent or routine symptomatic referral, and ~30% are diagnosed from screening [5].

We reported previously on breast cancer diagnostic services during the first 6 [6] and 15 months [7] of the pandemic after March 2020. We have updated these findings using both pre-existing and newer publicly available data sources. Cancer Waiting Time (CWT) data are published monthly and report measures of cancer service activity and performance including counts of referrals and first treatments [8]. The NHSBSP annual report details the number of cancers diagnosed through routine screening [9]. During the pandemic, important new resources were introduced to provide contemporaneous data to assist the recovery of cancer services. Information about stage at diagnosis is recorded in the COVID-19 Rapid Cancer Registration and Treatment Data (RCRD) datasets, and the COVID-19 Cancer Equity data packs, a subset of the CWT data, report referral and first treatment activity for each tumour type by age [10].

In the early part of the COVID-19 pandemic, from March 2020, all breast cancer screening units in England paused routine screening invitations. Services were resumed from July 2020, and all breast screening units reported sending routine invitations by September 2020. Symptomatic services were not suspended, but there was a large decrease in referrals following the first lockdown in March 2020, which largely recovered by August 2020. Referral rates remained at least at pre-pandemic levels during the further lockdowns [6, 7].

We report key measures of breast cancer diagnostic service activity, including the numbers of referrals, first treatments, number of screen-detected diagnoses, and the stage distribution of invasive breast cancer for the 3 financial years (April–March) of 2019/20, 2020/21 and 2021/22 (Table 1).

**Table 1:** The number of referrals, first treatments, screen-detected cancers and stage at diagnosis for breast cancer in England for March–April 2019/20, 2020/21, and 2021/22.

	Year		
	2019/2020	2020/2021	2021/2022
	% (*N*)	% (*N*)	% (*N*)
^a^Number of referrals	609,664	552,993	667,136
Urgent referrals	71.2 (433,880)	76.8 (424,970)	76.9 (512,782)
Routine referrals	28.8 (175,784)	23.2 (128,023)	23.1 (154,354)
^a,b^Number of first treatments	49,050	37,770	49,963
<50 years	16.3 (8012)	18.6 (7027)	14.3 (7156)
50–69 years	49.0 (24,037)	46.6 (17,683)	51.9 (25,945)
70+ years	34.7 (17,001)	34.6 (13,060)	33.7 (16,862)
^c^Number of screen-detected cancers	17,714	10,835	Awaited
^d^Number of invasive cancers	45,560	37,309	47,493
Stage 1	32.5 (14,829)	27.6 (10,296)	30.6 (14,543)
Stage 2	30.7 (13,969)	32.6 (12,170)	30.5 (14,486)
Stage 3	7.7 (3524)	8.3 (3105)	6.7 (3163)
Stage 4	3.1 (1390)	3.9 (1451)	3.2 (1522)
Unknown	26.0 (11,848)	27.6 (10,287)	29.0 (13,779)

Data shown are publicly available at:

^a^Cancer Waiting Time data at NHS England https://www.england.nhs.uk/statistics/statistical-work-areas/cancer-waiting-times/

^b^COVID-19 Cancer Equity data packs http://www.ncin.org.uk/local_cancer_intelligence/cadeas#covid-19

^c^Breast Screening Programme https://digital.nhs.uk/data-and-information/publications/statistical/breast-screening-programme

^d^COVID-19 Rapid Cancer Registration and Treatment Data https://www.cancerdata.nhs.uk/covid-19/rcrd

The overall number of referrals was 9% lower in 2020/21 and 9% higher in 2021/22 compared to 2019/20. However, the patterns of referrals changed and the proportion of patients referred urgently was higher during the first two years of the pandemic than 2019/20. There were ~10,000 fewer urgent referrals and ~48,000 fewer routine referrals in 2020/21 compared to 2019/20. The proportion of referrals that resulted in a cancer diagnosis (the cancer conversion rate) was similar for the two years (5.5% in 2019/20 and 5.7% in 2020/21 for urgent and 1.3% for routine referrals in both years) [10]. The data for conversion rates for 2021/22 are awaited and should clarify whether the observed increase in urgent referrals in the second year of the pandemic has led to an increased number of women diagnosed with breast cancer through this route.

The first treatment counts are a surrogate measure of the number of new cases of invasive and non-invasive breast cancer from all routes to diagnosis, including the NHSBSP. In 2019/20, the year before the pandemic, the number of first treatments for breast cancer was 49,050. This number was 23% lower in 2020/21 and 2% higher in 2021/22. The proportion of cancers diagnosed in women aged 70+ years remained roughly a third throughout the pandemic. Among patients aged 50–69 years, there was a 26% fall in the number of new diagnoses in 2020/21 and an 8% increase in 2021/22, compared to 2019/20. This is likely to be related to the pause and later resumption of NHSBSP invitations, as 60% of patients diagnosed with breast cancer in this age group are diagnosed within the NHSBSP.

Assuming 2019/20 was a ‘normal’ year for the number of breast cancer diagnoses, these data suggest that there may be ~10,300 “missing” women with breast cancer since the start of the pandemic. The NHSBSP data show 6879 fewer women were diagnosed through screening in 2020/21 compared to 2019/20 and the data for 2021/22 are awaited. This reduction in numbers of screen detected cancer may account for roughly two-thirds of these missing breast cancer diagnoses. It will remain to be seen how many of these women will be diagnosed with symptomatic breast cancer in the interval between screens, or whether they will be diagnosed following their next routine screening invitation.

We show the stage distribution for invasive breast cancer for these three years of interest as reported in the RCRD, which contains proxy tumour registrations for invasive breast cancer only and is produced approximately 4 months after a diagnosis and has high proportions of missing stage data [10]. In comparison, the gold standard patient level cancer registration data usually takes about 20 months to produce and is now largely complete for stage data [11]. The RCRD data suggest a reduction in the proportion of stage 1 breast cancers diagnosed in 2020/21, which again likely reflects the pause in screening as roughly 68% of patients with screen detected cancers have stage 1 cancer at diagnosis, compared to 31 and 42% following urgent or routine referral [12].

A recent study has estimated that the pandemic related disruption to the NHSBSP may result in 148–687 additional breast cancer deaths but scale of the impact ultimately depends on the speed of recovery [13]. Also of potential concern, are the apparently higher proportions of patients with locally advanced and metastatic disease in 2020/21 compared to the previous and subsequent years of interest. The fully validated patient level NCRAS data will be needed to rigorously quantify any stage shift.

Collectively, these data suggest that breast cancer diagnostic service activity has subsequently equalled or surpassed pre-pandemic activity levels which is reassuring. The observed annual increase in the proportion of patients with symptoms referred urgently will present capacity challenges to service providers. The impact of the COVID-19 pandemic on longer-term outcomes remains unclear.

## Data Availability

All the data used in this study are in the public domain and accessible at https://www.england.nhs.uk/statistics/statistical-work-areas/cancer-waiting-times/; http://www.ncin.org.uk/local_cancer_intelligence/cadeas#covid-19; https://digital.nhs.uk/data-and-information/publications/statistical/breast-screening-programme; https://www.cancerdata.nhs.uk/covid-19/rcrd.
